# A 2-year observational study of patients with relapsing-remitting multiple sclerosis converting to glatiramer acetate from other disease-modifying therapies: the COPTIMIZE trial

**DOI:** 10.1007/s00415-014-7446-0

**Published:** 2014-08-14

**Authors:** Tjalf Ziemssen, Ovidiu A. Bajenaru, Adriana Carrá, Nina de Klippel, João C. de Sá, Astrid Edland, Jette L. Frederiksen, Olivier Heinzlef, Klimentini E. Karageorgiou, Rafael H. Lander Delgado, Anne-Marie Landtblom, Miguel A. Macías Islas, Niall Tubridy, Yossi Gilgun-Sherki

**Affiliations:** 1 Neurologische Universitätsklinik, Klinikum Carl Gustav Carus, Fetscherstraße 74, 01307 Dresden, Germany; 2 Carol Davila University of Medicine and Pharmacy, Bucharest, Romania; 3 Hospital Britanico de Buenos Aires, Buenos Aires, Argentina; 4 Virga Jessaziekenhuis, Hasselt, Belgium; 5 Hospital de Santa Mari, Hasselt, Belgium; 6 Central Hospital of Buskerud, Drammen, Norway; 7 Glostrup Hospital, University of Copenhagen, Glostrup, Denmark; 8 Tenon Hospital, Paris, France; 9 General Hospital of Athens, Athens, Greece; 10 Department of Neurology and Department of Clinical and Experimental Medicine, University of Linköping, Linköping, Sweden; 11 Department of Medical Specialists and Department of Medicine and Health Sciences, Linköping University, Motala, Sweden; 12 Central University of Guadalajara, Guadalajara, Jalisco Mexico; 13 School of Medicine and Medical Science, Dublin University, Dublin, Ireland; 14 Teva Pharmaceuticals Industries Ltd, Petach Tikva, Israel

**Keywords:** Disease-modifying therapy, Glatiramer acetate, Multiple sclerosis, RRMS

## Abstract

Studies suggest that patients with relapsing-remitting multiple sclerosis (RRMS) who do not benefit from other disease-modifying treatments (DMTs) may benefit from converting to glatiramer acetate (GA). COPTIMIZE was a 24-month observational study designed to assess the disease course of patients converting to GA 20 mg daily from another DMT. Eligible patients had converted to GA and had received prior DMT for 3–6 months, depending on the reasons for conversion. Patients were assessed at baseline and at 6, 12, 18, and 24 months. In total, 672 patients from 148 centers worldwide were included in the analysis. Change of therapy to GA was prompted primarily by lack of efficacy (53.6 %) or intolerable adverse events (AEs; 44.8 %). Over a 24-month period, 72.7 % of patients were relapse free. Mean annual relapse rate decreased from 0.86 [95 % confidence interval (CI) 0.81–0.91] before the change to 0.32 (95 % CI 0.26–0.40; *p* < 0.0001) at last observation, while the progression of disability was halted, as the Kurtzke Expanded Disability Status Scale (EDSS) scores remained stable. Patients improved significantly (*p* < 0.05) on measures of fatigue, quality of life, depression, and cognition; mobility scores remained stable. The results indicate that changing RRMS patients to GA is associated with positive treatment outcomes.

## Introduction

Multiple sclerosis (MS) is a chronic, progressive, autoimmune diffuse inflammatory disease of the central nervous system [1]. Historically, the disease and the efficacy of MS treatments were measured by the extent to which clinical progression was slowed or halted, using relapse rates or the progression of disability [2, 3]. However, we now know that other considerations must also be taken into account, including fatigue, quality of life (QoL), etc. [4]. At least 30 % of patients show a suboptimal response to first-line disease-modifying treatments (DMTs) for relapsing-remitting multiple sclerosis (RRMS) during the first year of treatment [5]. There are no acceptable criteria to guide physicians when converting from one first-line DMT to another, and such decisions are generally based on the physician’s judgment.

Studies have shown that the three most common reasons why physicians in clinical practice convert an MS patient’s medication are lack of efficacy or suboptimal response, [5, 6] intolerable drug-induced adverse events (AEs) [7, 8], and the development of neutralizing antibodies [9–11], which are known to block the biological activity of interferon (IFN) and natalizumab therapy [12]. It has been suggested that clinical observations such as relapse rate and disability or findings of magnetic resonance imaging (MRI) may be used to define criteria for converting from one DMT to another in clinical practice [12, 13]. One study analyzed whether the first relapse and time from the first to second relapse would be able to predict treatment failure [14]. However, none of these criteria has proved useful in determining whether a patient would benefit from a treatment change.

Converting therapy within the IFN-β class may not always benefit the patient [15]. Patients who present with neutralizing antibodies during IFN treatment do not benefit from converting from one IFN to another or from continuous therapy with any subcutaneous IFN-β preparation [15]. Conversely, studies have demonstrated that there is a clinical benefit in changing either from one class of first-line DMT to another or to second-line treatments [13, 16–18]. With some DMTs (e.g. natalizumab, which is indicated for patients for whom IFN therapy has not been effective), the use of escalating doses has been proven to improve efficacy compared with converting to another DMT [19]. However, despite its efficacy profile, the safety and tolerability of natalizumab are a concern because of the risk of progressive multifocal leukoencephalopathy (PML) [20].

The copolymer glatiramer acetate (GA; Copaxone) is approved as a 20-mg daily subcutaneous (s.c.) injection for reducing relapse frequency in patients with RRMS [21]. Post-marketing experience with GA includes more than 1.88 million patient-years of exposure and, in some patients, more than 20 consecutive years of treatment [22]. Two prospective open-label studies have shown a beneficial effect of GA for subjects who did not benefit from previous sequential IFN treatment, either because of lack of perceived clinical effects or AEs [13, 17]. The COPTIMIZE trial was designed to provide insight into patients’ outcomes and attitudes toward converting to GA when another DMT is ineffective or intolerable. This also allowed investigators to assess the impact of the mild adverse effect profile of GA, which differs in many ways from other DMTs, and the positive impact of GA on QoL parameters [23–25].

## Methods

### Study design

COPTIMIZE was a 2-year international, multicenter, prospective, non-interventional, longitudinal, and observational study conducted in 148 study centers across 19 countries. Included were patients who had converted from another DMT to GA 20 mg daily within 3–6 months of screening.

An electronic case report form (eCRF) was completed by attending neurologists (investigators) to assess the disease course and rationale for converting treatment to GA. Data were collected by means of standardized eCRF on a password-protected website, at baseline and then at 6-month intervals for a total of five data collection time points over 24 months. Baseline assessment included patients’ demographic characteristics, MS disease history, reasons for changing medication, annualized relapse rate (ARR) in the 2 years before the conversion, expanded disability status scale (EDSS)/mobility score measured within 2 years before the conversion and at recruitment, MRI data, cognitive functions by Paced Auditory Serial Addition Test (PASAT) [26], and impact of fatigue on daily activities by Modified Fatigue Impact Scale (MFIS; the effects of fatigue on physical, cognitive, and psychosocial functioning) [27]. Patients answered 21 questions on fatigue severity, with scores ranging from ‘never’ (0) to ‘highly’ (4), which denotes severe fatigue.

Assessments at 6-month intervals included relapses within the previous 6 months and the EDSS/mobility score; the EDSS assessment was performed via the Neurostatus *e* test [28]. Confirmed progression (i.e. worsening of the EDSS from baseline to final examination) was defined as an increase of one point if the baseline EDSS score was between 0 and 5, and by an increase of 0.5 points if the baseline score was >5.0. Changes in function were assessed by the Functional Assessment of Multiple Sclerosis (FAMS) [29]. Scores on the FAMS range between 0.00 and 176.00 points, with an increase in score indicating an increase in functional abilities. Depression was measured by the Center for Epidemiological Studies Depression Scale (CES-D) [30]. CES-D scores ranged from 0 to 60 points, with higher scores indicating more symptoms of depression during the past week.

### Patients

To be included, patients had to have a diagnosis of RRMS, to have converted to GA within 3 months before recruitment, and to have available ARR and EDSS data acquired in the year before inclusion. Patients could have been treated with any DMT for up to 6 months before the treatment conversion, if the change was due to unverified drug inefficacy or AEs.

Patients were classified based on their individual premedication: ‘de novo’ patients had not received any pharmaceutical MS medication, ‘converter’ patients had received another kind of DMT before recruitment, and ‘post-chemotherapy’ patients had received chemotherapeutic medication before recruitment.

### Study endpoints

The primary study endpoint was disease course of subjects converted from one DMT class (IFN) to another (GA) as measured by ARR before and after the conversion, annualized rate of deterioration (ARD: rate of deterioration as measured by mean EDSS), and mobility score in the year before and following the change to GA.

Secondary endpoints included reasons for changing DMT; characteristics of patients failing to benefit from previous DMT; QoL changes measured by FAMS following GA conversion; impact of fatigue on daily activities, measured by the MFIS; change in rates of depression as evaluated by CES-D; and changes in AEs before and after the conversion to GA.

This study was conducted in accordance with the 18th World Medical Assembly (Helsinki) recommendations and amendments, as well as guidelines for Good Epidemiology Practice. Patients’ personal data and investigator data included in the sponsor database were treated in compliance with all local applicable laws and regulations.

### Statistical analyses

The intention-to-treat cohort, consisting of all enrolled subjects who took at least one dose of GA, was used for all efficacy and safety assessments. Descriptive procedures were used to represent data. Tests of significance (signed rank test and binomial test) were used to measure changes in efficacy parameters from baseline to final examination. Wilcoxon signal rank was used within groups for EDSS, MFIS, QoL, CES-D, and PASAT (excluding ARR). Kruskal–Wallis was used between groups for EDSS. Poisson regression within and between groups was used for ARR. ARR and ARD before and after the conversion was analyzed using repeated measures analysis of covariance using the maximum likelihood ratio. Log transformation was implemented to the ARR and ARD to establish if there was a significant deviation of ARR and ARD from normality (i.e. if *p* < 0.001 on the Shapiro–Wilk test).

## Results

### Patient disposition

A total of 672 patients were enrolled in the study. Data on 555 patients (82.6 %) were available at 365 days, and data on 423 (63.0 %) were available at 730 days. The mean duration of observation was 594.7 days [±standard deviation (SD) = 221.3] in 634 patients who had one or more examinations. Table 1 details patient demographics and disease characteristics.

**Table 1:** Baseline demographics and disease characteristics

Characteristics	Patients with data	Overall
Female gender, *n* (%)	672	476 (70.8)
Mean age, years (SD)	672	39.9 (10.2)
Mean duration of disease since onset, mo (SD)	615	97.2 (78.9)
Mean time since MS diagnosis, mo (SD)	632	69.7 (61.3)
Median ARR measured over the past 2 years before GA (SD)	625	0.86 (0.67)
Distribution of patients by ARR range, *n* (%)	660	
<1		329 (49.9)
≥1 and <3		318 (48.2)
≥3		13 (2.0)
Clinical type of MS, *n* (%)	657	
RRMS with incomplete remissions		264 (40.2)
RRMS with complete remission		383 (58.3)
Clinically isolated syndrome		1 (0.2)
Other		9 (1.4)
Mean EDSS score measured over the past 2 years before GA (SD)	878	2.8 (1.7)
Mean EDSS score at time of conversion (SD)	600	3.0 (1.9)
Mobility score, *n* (%)	595	
Asymptomatic		111 (18.7)
Able to walk unaided >500 m		336 (56.5)
Able to walk unaided for <500 m		60 (10.1)
Walking with unilateral support		51 (8.6)
Walking with bilateral support		22 (3.7)
Need of wheelchair outdoors		15 (2.5)
MRI data available, *n* (%)	672	193 (41.0)

*ARR* annualized relapse rate, *EDSS* Expanded Disability Status Scale, *GA* glatiramer acetate, *MRI* magnetic resonance imaging, *MS* multiple sclerosis, *RRMS* relapsing-remitting multiple sclerosis, *SD* standard deviation

### Baseline demographics and patient classification

Demographics and disease characteristics are shown in Table 1. Of the 672 patients enrolled, 640 (95.2 %) were classified as ‘converter’ patients (had received other DMT before enrollment), and the efficacy analysis was restricted to these patients. Nine (1.3 %) were classified as ‘post-chemotherapy’ patients, and 23 patients (3.4 %) were missing classification data. In converted patients, a change of therapy to GA was prompted primarily by lack of efficacy (343/640; 53.6 %) or intolerable AEs (287/640; 44.8 %), caused by the corresponding premedication. [Note: The number of patients who changed to GA due to a lack of efficacy (343) and the number that changed due to AEs (287) sums to 630, not 640, as there are multiple reasons aside from these two that were cited by patients for changing therapy]. In the majority of converted patients (553/640; 86.4 %), only a single DMT agent had been used before the conversion to GA therapy. Eighty patients (12.5 %) had received two DMT agents, and six patients (0.9 %) had received three DMT agents before the change to GA. One patient (0.2 %) was missing information on number of prior DMT treatments received.

**Fig. 1:**
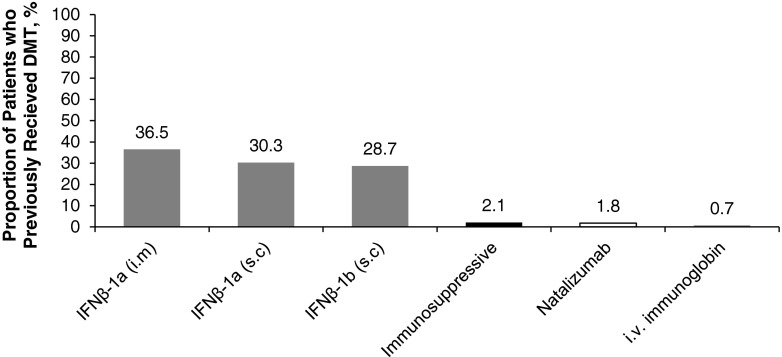
Type of disease-modifying therapy used by patients before converting to glatiramer acetate in patients with previous type known (*n* = 617). *IFN* interferon, *i.m* intramuscular, *i.v.* intravenous, *s.c* subcutaneous

Of the patients converted, documentation on type of DMT was available for 617 patients and missing for 23 patients. Most patients converted (589/617) (95.5 %) had received IFN-β before converting (Fig. 1).

Table 2 details the baseline disease characteristics of those patients who were converted because of lack of efficacy or because of AEs. The clinical type of disease, disease activity over the past 2 years, and the decision to convert were significantly different between these two groups. A greater proportion of patients who converted because of lack of efficacy presented with RRMS with incomplete remissions, while most who converted because of AEs had RRMS with complete remissions. Exacerbations tended to be rare in both groups. However, exacerbations of disease were more frequent in those who were converted because of lack of efficacy, while stable disease was more prominent in those who were converted because of AEs. While, in most cases, the decision to convert was made solely by the patient’s physician, a mutual decision was more common among the patients who converted because of AEs (Table 2). The majority of patients who converted because of AEs discontinued IFN therapy because of flu-like symptoms [180/287 (62.7 %); Table 3].

**Table 2:** Disease characteristics of patients converted to glatiramer acetate because of lack of efficacy or adverse events (*n* = 630)

Characteristics	Lack of efficacy (*n* = 343)	Adverse events (*n* = 287)	*p* value
Clinical disease type over the past 2 years, %			<0.0001
RRMS with complete remissions	30.1	50.3	
RRMS with incomplete remissions	67.5	49.3	
Other	2.4	0.5	
Activity of disease over the past 2 years, %			<0.0001
Stable MS	8.9	23.7	
Exacerbations rare (<1 relapse/year)	35.9	47.3	
Slow progression (< 1 point increase in EDSS in the last year)	16.1	9.4	
Frequent exacerbations (≥1 relapse/year)	31.5	16.3	
Fast progression (≥1 point increase in EDSS in the last year)	3.2	1.0	
Could not be classified	4.4	2.5	
Decision to convert therapy made by, %			<0.0001
Physician	86.2	59.9	
Patient	2.8	7.4	
Both	10.9	32.7	

*EDSS* Expanded Disability Status Scale, *MS* multiple sclerosis, *RRMS* relapsing remitting MS

**Table 3:** Reasons for discontinuing interferon treatment before study entry among patients converted to glatiramer acetate because of intolerable adverse events (*n* = 287)

Reason	Patients, *n* (%)
Flu–like symptoms	180 (62.7)
Subjective	83 (28.9)
Skin reactions	51 (17.8)
Blood work	29 (10.1)
Others	64 (22.3)
Not specified	2 (0.7)

Patients responded with up to three possible reasons

Among the nine patients classified as being ‘post-chemotherapy’, the most common reasons for converting were worsening of EDSS (*n* = 7) and severity of relapses (*n* = 4), followed by high lesion load on MRI (*n* = 2) and a high relapse rate (*n* = 1). Multiple reasons for converting could be recorded for a single patient. All nine patients had undergone escalation therapy, seven had received mitoxantrone, one had received cyclophosphamide, and one cyclophosphamide followed by IFN.

### Efficacy of GA

#### ARR

**Fig. 2:**
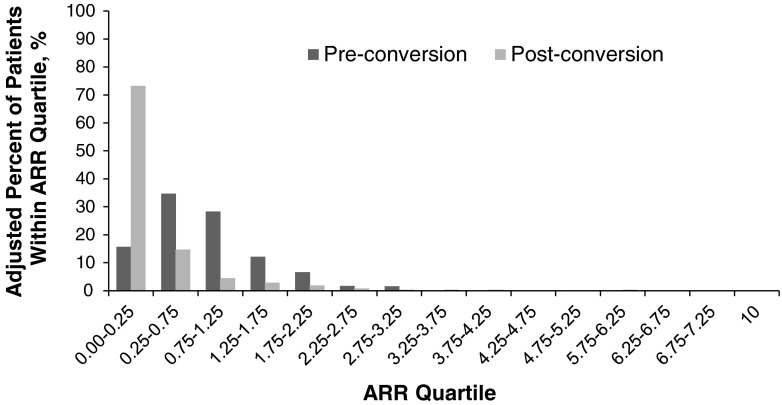
Distribution of annualized relapse rates (ARRs) before and during glatiramer acetate therapy (*n* = *625*). Patients with a very high annualized relapse rate terminated glatiramer acetate treatment after a short period of observation because of relapses

**Fig. 3:**
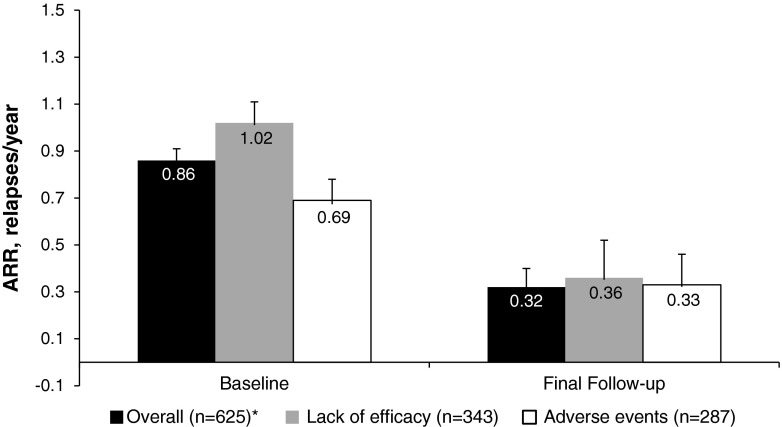
Change in annualized relapse rate (ARR) in all patients receiving glatiramer acetate (GA) therapy (*n* = 625) and in patients with known reason for the conversion to GA (*Asterisk* denotes that the overall number of patients with ARR data does not equal the sum of the number of patients who converted to GA due to lack of efficacy and adverse events because of double counting of patients who reported both reasons for converting). All reductions in ARR within groups were statistically significant (*p* < 0.0001)

Data on ARRs before converting to GA and during the study were available for 625 patients. The majority of these patients [*n* = 458/625 (73.3 %)] experienced less than 0.25 relapses/year while receiving GA therapy (Fig. 2). Overall, patients experienced a significant reduction in the mean number of relapses from baseline while on GA therapy from 0.86 to 0.32 (mean change −0.54; *p* < 0.0001 Chi squared; Fig. 3). Reductions in ARR from baseline were significant regardless of whether patients converted because of lack of efficacy or AEs (mean change −0.66 and −0.36, respectively; *p* < 0.0001 in both groups; Fig. 3). However, the decrease in ARR was significantly greater in patients converting for lack of efficacy versus AEs (*p* = 0.0021).

#### Confirmed EDSS change

**Fig. 4:**
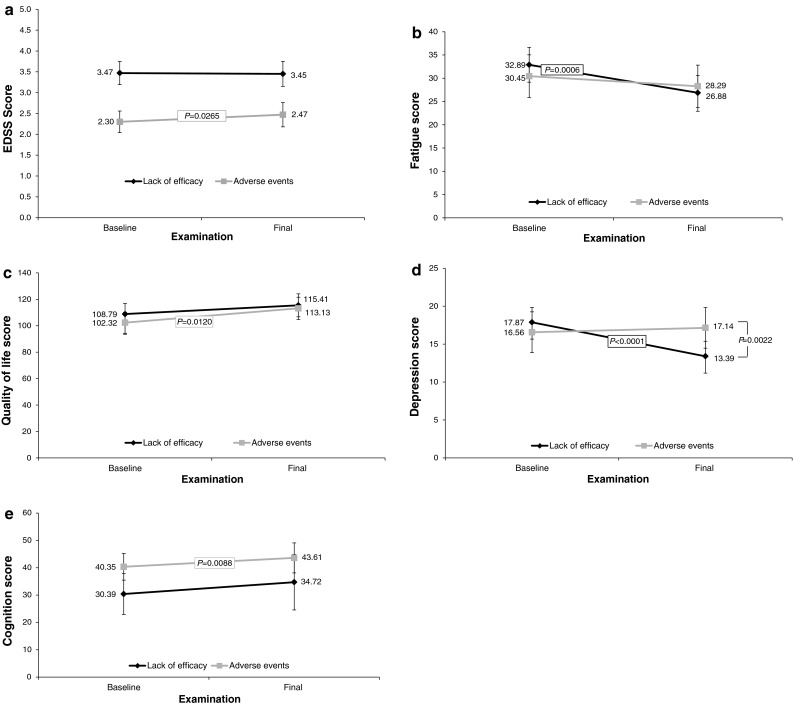
**a** Change in Expanded Disability Status Scale (EDSS) score in patients converted to glatiramer acetate, by the reason for the conversion. **b** Change in fatigue score (Modified Fatigue Impact Scale) in patients converted to glatiramer acetate, by the reason for the conversion. **c** Change in quality of life score (Functional Assessment of Multiple Sclerosis) in patients converted to glatiramer acetate, by the reason for the conversion. **d** Change in depression score (Center for Epidemiological Studies Depression Scale) in patients converted to glatiramer acetate, by the reason for the conversion. **e** Change in cognition score (paced auditory serial addition test) in converting patients by the reason for the conversion

Data on 399 patients with at least one confirmed EDSS progression after baseline examination were evaluated. The proportion of patients without confirmed progression (343/399 patients, 86.0 %) was significantly higher than with confirmed progression (56/399 patients; 14.0 %; *p* < 0.0001, binominal-test with *H*_0_ proportion = 50 %). When analyzed by reason for conversion only, patients who converted because of intolerable AEs had a significant increase in EDSS from baseline (+0.17; *p* = 0.0265, Fig. 4a) but there was no significant difference between the values in the two groups.

#### Mobility score

A total of 542 patients had at least one mobility score after the baseline examination. The majority of patients (*n* = 348; 64.2 %) did not experience any worsening in mobility A total of 103 (19.0 %) patients reported better mobility, while 91 (16.8 %) reported worse mobility. The difference in the numbers of patients showing improvement or worsening was not significant by binominal test with *H*_0_ proportion = 50 % (*p* = NS).

#### Impact on fatigue

Data on 287 patients with MFIS scores were available for evaluation. Overall, mean MFIS scores decreased significantly from baseline to final examination, with a difference of −3.59 points ±15.05, *p* < 0.0001; Table 4. The reduction in fatigue was greater in patients who converted because of lack of efficacy (−6.01 points from baseline; *p* = 0.0006), compared with those who converted because of AEs (−2.16 points; *p* = NS; Fig. 4b).

**Table 4:** Change in secondary efficacy endpoints from baseline to final observation of all patients irrespective of previous treatment or reason for conversion

Scale, mean (95 % CI)	Patients with data (*n*)	Baseline	Final	*p* value
Fatigue, MFIS	287	31.94 (29.67–34.22)	28.36 (26.00–30.72)	<0.0001
Quality of life, FAMS	218	102.67 (97.78–107.57)	108.61 (103.43–113.80)	0.0227
Depression, CES-D	299	16.13 (14.85–17.40)	14.63 (13.38–15.88)	0.0111
Cognition, PASAT	72	37.46 (33.93–40.99)	41.75 (37.79–45.71)	<0.0001

*CES*-*D* Center for Epidemiological Studies Depression Scale, *CI* confidence interval, *FAMS* Functional Assessment of Multiple Sclerosis, *MFIS* Modified Fatigue Impact Scale, *PASAT* paced auditory serial addition test

#### Change in QoL

A total of 218 patients had available QoL data at baseline and at the final examination.

A significant improvement in QoL score of 5.94 (±31.57; *p* = 0.0227) from baseline to final examination was reported (Table 4). QoL improved regardless of the reasons for treatment conversion. Greater improvement was observed in patients who converted because of AEs (+10.81 points from baseline; *p* = 0.0120), compared with those who converted because of lack of efficacy (+6.62 points; *p* = NS; Fig. 4c).

#### Depression

Data on 299 patients were available for evaluation. There was a significant improvement overall in the depression score following the conversion to GA therapy (−1.50 ± 10.84 from baseline; Table 4). Improvement was most commonly observed in patients who were converted because of lack of efficacy (−4.48 points from baseline; *p* < 0.0001). No improvement was reported in those who converted because of AEs (+0.58 points; *p* = NS; Fig. 4d).

#### Cognition changes

In the 72 patients for whom cognition (PASAT) data were available, scores improved by a mean of 4.29 ± 9.28 (*p* < 0.0001; Table 4). Improvement in cognition was observed in patients who converted because of AEs (+3.26 points from baseline; *p* = 0.0088), as well as in those who converted because of lack of efficacy (+4.33 points from baseline; *p* = NS; Fig. 4e). Both baseline and final scores were notably higher in the group that converted because of AEs (Fig. 4e).

#### Patient reporting of efficacy

Among the 660 patients with available data, only 49 patients (7.4 %; *p* < 0.0001) reported that GA was less effective than their previous DMT, while significantly more patients [348 (52.7 %)] reported that GA treatment was more effective than their previous DMT, and 263 patients (39.9 %) reported no difference.

### Safety and tolerability

A total of 196 AEs occurred in 104 patients [15.5 % of all patients (*n* = 672)], with the majority of events deemed probably [104 events in 56 patients (8.3 %)] or possibly related to GA therapy [45 events in 26 patients (3.8 %)]. Most common AEs by preferred term and system organ class are shown in Table 5 in addition to AEs by severity reported. 174 of all 672 patients (25.9 %) terminated GA treatment during the observation period. Table 6 details the physician- and patient-reported reasons for termination.

**Table 5:** Most frequently reported adverse events by preferred term and by system organ class (*n* = 672)

Adverse events	Patients, *n* (%)	Number of events
Total reported adverse events	104 (15.5)	196
By system organ class (frequency of cases ≥3)		
General disorders and administration site conditions	50 (7.4)	78
Nervous system disorders	19 (2.8)	20
Skin and subcutaneous tissue disorders	18 (2.7)	21
Psychiatric disorders	11 (1.6)	14
Respiratory, thoracic and mediastinal disorders	10 (1.5)	12
Musculoskeletal and connective tissue disorders	8 (1.2)	9
Immune system disorders	7 (1.0)	7
Vascular disorders	7 (1.0)	7
Gastrointestinal disorders	5 (0.7)	6
Infections and infestations	3 (0.5)	3
Neoplasms benign, malignant, and unspecified	3 (0.5)	3
Adverse events by preferred term (frequency of cases ≥4)		
Injection-site pain	13 (1.9)	17
Injection-site reaction	10 (1.5)	10
Dyspnea	8 (1.2)	10
Depression	6 (0.9)	6
Hypersensitivity	6 (0.9)	6
Headache	5 (0.7)	5
Injection site induration	5 (0.7)	5
Lipoatrophy	5 (0.7)	5
Application site pain	4 (0.6)	4
Arthralgia	4 (0.6)	4
Fatigue	4 (0.6)	4
Flushing	4 (0.6)	4
Rash	4 (0.6)	4
Syncope	4 (0.6)	4
By severity		
Serious	7 (1.0)	10
Severe	18 (2.7)	32
Moderate	56 (8.3)	90
Mild	41 (6.1)	61
Not reported	9 (1.3)	13
Most common severe adverse events		
Injection-site pain	3 (0.5)	4
Dyspnea	4 (0.6)	4
Most common moderate adverse events		
Injection-site pain	6 (0.9)	8
Depression	6 (0.9)	6
Injection-site reaction	6 (0.9)	4
Hypersensitivity	6 (0.9)	4
Most common mild adverse events		
Injection-site pain	5 (0.7)	5
Injection-site reaction	5 (0.7)	5
Outcome of adverse events		
Ongoing at date of report	49 (7.3)	79
Completely resolved	48 (7.1)	84
Resolved with sequelae	8 (1.2)	12
Data missing	9 (1.3)	11
Unknown result	4 (0.6)	10
Action taken on Copaxone due to adverse events		
No action taken	71 (10.6)	128
Treatment permanently discontinued	31 (4.6)	46
Treatment temporarily interrupted	13 (1.9)	19
Data missing	2 (0.4)	2
Dose reduction	1 (0.2)	1
Patient assessment of adverse events		
Reported improvement after conversion to GA	430 (65.2)	N/A
Reported no change after conversion to GA	192 (29.0)	N/A
Reported feeling worse after conversion to GA	38 (5.8)	N/A

**Table 6:** Study termination and most commonly reported reasons for termination (*n* = 672)

Adverse events	Patients, *n* (%)
Patients discontinuing trial for any reason^a^	174 (25.9)
One reported reason	156 (23.2)
Two reported reasons	15 (2.2)
Three reported reasons	3 (0.5)
Most common physician-reported reasons	
Lack of efficacy or perceived efficacy	42 (6.3)
Loss to follow-up	40 (6.0)
Adverse events	31 (4.6)
Other	26 (3.9)
Most common patient-reported reasons	
Lack of efficacy or perceived efficacy	27 (4.0)
Consent withdrawn	21 (3.1)
Fear of adverse events	8 (1.2)

^a^Patients cited up to three reasons for discontinuing treatment, explaining why the number of total reported reasons for discontinuation (195) exceeds number of discontinuing patients (174)

## Discussion

It is reported that patients with RRMS frequently convert DMT because their original therapy is not optimally effective or produces intolerable AEs [6, 7, 9, 12, 17–19]; those were the main reasons for converting to GA therapy in this study. Depending on the reasons for converting, lack of efficacy or adverse reactions, patients may have a greater or lesser response to the new agent.

ARR is an important indication of the inflammatory component of MS. In the COPTIMIZE study, ARR was significantly reduced from baseline after converting to GA, both in patients who converted because of lack of efficacy and those who converted because of AEs.

No significant changes in EDSS scores were observed in patients who were converted to GA therapy. However, it is important to note that following the conversion to GA, a greater proportion of patients had no confirmed progression, as measured by the EDSS; only modest changes in EDSS scores from baseline were observed. Subgroup analysis revealed EDSS scores to be higher at both baseline and final examination among those converted because of lack of efficacy rather than AEs.

As our understanding of MS improves, it has become clearer that symptoms beyond disability scores, such as EDSS, are important [23]. For example, fatigue in MS has been correlated not only with neurodegenerative processes that cause functional reorganization resulting in increased metabolic demands [31, 32], but also, recently, with disturbance in central neuronal pathways [33]. Interestingly, in one study, patient-reported fatigue was observed to significantly improve after converting to GA, consistent with previous reports of increased improvement of fatigue symptoms with GA use [26, 34]. Similarly, cognition, correlating with cortical atrophy in MS patients [35], improved significantly from baseline after converting to GA. Cognitive symptoms in MS have been associated with cortical atrophy [35], but it is important to remember that such symptoms in many patients can vary over time and be a result of fatigue. Of course, patients who improve after switching from IFN to GA may do so because they no longer experience the typical flu-like side effects that increase fatigue. Patients also improved significantly on measures of QoL and depression, while mobility scores remained stable. Improvements in QoL were more pronounced in those who converted because of AEs versus lack of efficacy, which is consistent with intolerable AEs, which significantly impact QoL, being eliminated or reduced after conversion to GA.

Depression scores significantly improved during GA treatment in patients who converted due to lack of efficacy, but were unchanged in those who converted because of AEs. Improved scores in patients previously experiencing a lack of efficacy may have been due to a heightened confidence in the ability of their new regimen to slow disease progression.

Taken together, the decrease in ARR and lack of EDSS progression observed in patients who converted to GA therapy represent significant real-world improvements attained by RRMS patients whose disease was not adequately controlled by their previous DMT. This is an important finding, because it points to the ability of GA to modify patient progression on a real-world level, as measured by clinical relapses and other patient-reported outcomes, including fatigue and depression.

Of the 672 patients included in the study, AEs occurred in 15.5 % of patients. These AEs were mainly attributable to injection-site reactions or pain. Because neurodegenerative activity is observed in MS patients even in early stages of the disease [36], it is important to establish rigorous algorithms to optimize treatment in those responding sub-optimally to their original therapy. Should monotherapy not prove optimal, combining a DMT with another treatment could provide an additive effect to control disease progression [37].

This observational study reflects both the limitations and advantages inherent in such a study design. Regression to the mean has been shown to be a common occurrence in longitudinal studies of MS patients with high levels of disease activity and may present a limitation in the present study given that there is no comparison to a matched control cohort [38]. This phenomenon would suggest that patients switching to GA as a consequence of the limited efficacy of prior therapy will tend to return to the average disease state over time, potentially accounting for reductions in the ARR rate. Other limitations include the potential for information or classification bias [39]. However, well-designed observational studies with appropriate statistical techniques provide valuable information, with high generalizability. Further, the overall sample size was relatively small, and sample sizes were not consistent throughout the different assessments (i.e. the same number of patients may not have been examined for fatigue as for cognition, etc.). Thus, it was not possible to pool the patients across all parameters. Further, because of the observational nature of this study, there was a fairly high dropout rate and considerable variability in the availability of patient data for different endpoints. However, the dropout rate included not only patients who left the study but also patients who had to be excluded from the study because of missing data from the participating sites. Nevertheless, the results of observational studies can be used to demonstrate real-world clinical outcomes, including improvement to patients’ daily lives, and fewer relapses and improved quality of life.

Despite study limitations, our observations corroborate the results of previous studies in which improved treatment response (i.e. reduced ARR, delayed disease progression) was observed in patients who converted from one DMT to another [5, 6, 14, 40–43]. Our findings also emphasize the importance of changing a therapeutic regimen to improve patients’ well-being (i.e. QoL, depression, fatigue) and control disease progression while overcoming treatment-related barriers (i.e. intolerable AEs) that could compromise compliance among patients responding sub-optimally to their current regimen and result in further disease progression.

Patients whose disease is progressing on their current DMT need to be converted in a timely manner. Future clinical trial designs should include patients converting from one DMT to another as a study arm. These trials could contribute to the development of consensus statements, treatment algorithms, and clinical parameters for changing treatment. This is important, as converting treatments is not always necessary and is associated with significant healthcare costs.
